# A Rare Cause of Persistent Pulmonary Hypertension Resistant to Therapy in The Newborn: Short-Rib Polydactyly Syndrome

**DOI:** 10.1155/2015/274639

**Published:** 2015-05-20

**Authors:** Nihat Demir, Erdal Peker, İbrahim Ece, Sultan Kaba, Kemal Ağengin, Oğuz Tuncer

**Affiliations:** ^1^Department of Pediatrics, Division of Neonatology, Yuzuncu Yil University School of Medicine, 65080 Van, Turkey; ^2^Department of Pediatrics, Division of Cardiology, Yuzuncu Yil University School of Medicine, 65080 Van, Turkey; ^3^Department of Pediatrics, Division of Endocrinology, Yuzuncu Yil University School of Medicine, 65080 Van, Turkey; ^4^Department of Pediatric Surgery, Yuzuncu Yil University School of Medicine, 65080 Van, Turkey

## Abstract

Short-rib polydactyly syndrome is an autosomal recessively inherited lethal skeletal dysplasia. The syndrome is characterized by marked narrow fetal thorax, short extremities, micromelia, cleft palate/lip, polydactyly, cardiac and renal abnormalities, and genital malformations. In cases with pulmonary hypoplasia, persistent pulmonary hypertension of the newborn can develop. In this paper, we present a term newborn with persistent pulmonary hypertension of the newborn, which has developed secondary to short-rib polydactyly syndrome and was resistant to therapy with inhaled nitric oxide and oral sildenafil.

## 1. Introduction

Short-rib polydactyly syndrome (SRPS) is an autosomal recessively inherited lethal skeletal dysplasia. The syndrome is generally classified as type 1 (Saldino-Noonan; OMIM 263530), type 2 (Majewski; OMIM 263520), type 3 (Verma-Naumoff; OMIM 263510), type 4 (Beemer-Langer; OMIM 269860), and the recently recognized type 5 (MIM 614091). Clinically, the syndrome is characterized by marked narrow fetal thorax, short extremities, micromelia, cleft palate/lip, polydactyly, cardiac and renal abnormalities, and genital malformation [1]. Furthermore, cone-shaped epiphyses, flat nose arc, thickening in the bile ducts, and clinical findings secondary to pulmonary hypoplasia can be rarely seen [2]. The marked narrow thorax and short ribs in SRPS can lead to pulmonary hypoplasia, and consequently both parenchymal hypoplasia and vascular hypoplasia in pulmonary hypoplasia can cause persistent pulmonary hypertension (PPH) [3]. In this paper, for the first time in the literature, we present a case with neonatal persistent pulmonary hypertension, which developed secondary to SRPS and which was resistant to therapy.

## 2. Case

**Figure 1:**
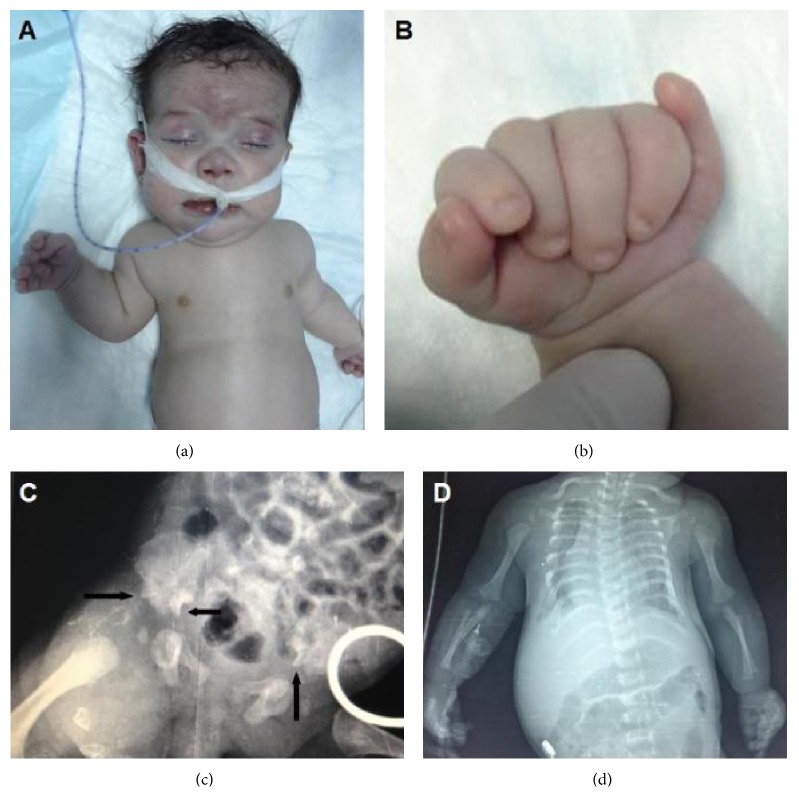
Clinical and X-ray features of the baby: (a) characteristic facial features of prominent forehead, low-set ears, narrow thorax, short upper extremities, a short and flat nose, and micrognathia; (b) polydactyly and hypoplastic nails of the hands; (c) trident aspect of the acetabular roof, ossification defects at the inferior aspect of the lateral iliac margin; (d) narrow thorax, short ribs, and high clavicle bones.

A 22-year-old healthy mother gave birth by cesarean section to a baby girl of 39-week gestational age. On her pregnancy follow-ups, obstetrical ultrasonography examination performed at the 20th gestational week (GW) demonstrated long tubular bones that were shorter than expected for GW, narrow thorax, and short ribs in the fetus. The mother and father were not relatives and did not have a history of a similar disorder. Right after birth, the newborn, who showed respiratory distress with respective Apgar scores of 5 and 7 at minutes 1 and 5, was transferred to the newborn intensive care unit, where she received intensive care supported by nasal continuous positive air pressure (CPAP). The newborn's birth weight measured 2780 (3–10th percentile); height was 46 cm (10th percentile), and head circumference was 34 cm (50th percentile). The findings on the physical examination of the newborn were wide open forehead, fallen ears, short and flat nasal arc, micrognathia, narrow thorax, short extremities, small epiglottis, micromelia, hypoplastic nails, and proximal polydactyly in both hands (Figures 1(a) and 1(b)). Her biochemical parameters and microbiological culture results were normal. Furthermore, the results of her transfontanelle and abdominal ultrasonography and cranial magnetic resonance examinations were normal. Her genetic analysis revealed 46,XY. Her direct radiogram showed narrow and low thorax with short horizontal costae, high clavicle bones (Figure 1(d)), dysplastic hypoplastic changes in the pelvic bones being more prominent particularly in the ileum, concave long bones, and endochondral ossification disorders (Figure 1(c)). With these clinical and radiological findings, the patient was diagnosed with SRPS. Upon worsening of her respiratory pattern (oxygenation index >30 and the mean airway pressure = 18 cm H_2_O), the patient was intubated and given mechanical ventilation support. The echocardiogram showed a clinically insignificant ventricular septal defect (VSD), patent ductus arteriosus (PDA), and pulmonary hypertension (pulmonary arterial pressure: 100 mm Hg). The situation was evaluated as pulmonary hypertension secondary to SRPS, and treatment with inhaled nitric oxide (iNO) (15–40 ppm/dose) and oral sildenafil (1 mg/kg/dose, 4 times a day) was begun. The patient was followed up by daily controls of pulmonary pressure and methemoglobin measurements. After one week, the pulmonary pressure of the patient could be brought down to 70 mm Hg. However, the general condition of the patient showed no improvement, and she was lost on day 15 of the iNO and oral sildenafil treatment.

## 3. Discussion

Short-rib polydactyly syndrome is one of the most lethal skeletal dysplasias, which is characterized by narrow thorax with short costae, shortness in long bones, and sometimes polydactyly [4]. One of the most important mortality determinants in SRPS is the degree of pulmonary hypoplasia caused by narrow thorax [5]. Congenital pulmonary hypoplasia (CPH) is seen in 1 : 1000 of the newborns and is either primary or secondary. The primary causes of CPH are genetic defects, scimitar syndrome, Down syndrome, and pterygium syndrome, whereas the secondary causes are congenital diaphragmatic hernia, neuromuscular disorders, deficiency of amniotic fluid in pregnancy (oligohydramnios), and disorders accompanied by poor pulmonary blood flow [6, 7]. Congenital pulmonary hypoplasia can present itself as respiratory distress, reduced breath sounds, normal or small-bell shaped thorax, or clinical findings of PPH [6]. The prevalence of persistent pulmonary hypertension of the newborn (PPHN), one of the most frequent causes of respiratory insufficiency in the newborn, is 0.4–0.6 : 1000 [8, 9]. Although PPHN can be caused by various factors, the most important known cause of this disorder is the insufficient fall in the pulmonary vascular resistance, which is normally expected to fall right after birth. Persistent pulmonary hypertension in the newborn usually accompanies meconium aspiration, transient tachypnea of the newborn, and respiratory distress syndrome [9]. However, it can rarely be associated with capillary dysplasia, genetic defect in surfactant synthesis, congenital abnormalities, and oligohydramnios. The prognosis in PPHN cases accompanied by pulmonary hypoplasia is quite poor. The high mortality rate in such cases is due to the underdevelopment of the pulmonary parenchyma and the vascular bed, as well as to the insufficient response to therapy [10]. Some recent studies have reported an association between PPHN and mutations in corticotropin-releasing hormone receptor 1 (CRHR1) and transmembrane protein 70 (TMEM70) [11, 12]. Rocha et al. [9] reported that the most frequent cause among their 78 patients with PPHN was pulmonary hypoplasia secondary to diaphragmatic hernia. They found diaphragmatic hernia in almost all of their patients. As we also found the same finding in our case, in particular the cases with pulmonary hypoplasia showed insufficient response to therapy [9]. The rare causes of PPHN are syndromes such as scimitar syndrome, Costello syndrome, Down syndrome, and pterygium syndrome [6, 7, 13]. A limited number of studies have reported that PPHN cases secondary to congenital diaphragmatic hernia give insufficient response to therapy, particularly to therapy with iNO [10]. The insufficient response to therapy in our case is compatible with this finding in the literature.

In conclusion, this case has been presented to underline that, in cases with SRPS, PPHN can develop dependent on the degree of thoracic narrowness and pulmonary hypoplasia and that PPHN can be resistant to therapy.
